# Effect of High Glucose Levels on White Adipose Cells and Adipokines—Fuel for the Fire

**DOI:** 10.3390/ijms18050944

**Published:** 2017-04-29

**Authors:** Alexander Sorisky

**Affiliations:** Chronic Disease Program, Ottawa Hospital Research Institute, and Departments of Medicine and of Biochemistry, Microbiology & Immunology, University of Ottawa, Ottawa, ON K1H 8L6, Canada; asorisky@ohri.ca; Tel.: +1-613-737-8899

**Keywords:** adipocytes, hyperglycaemia, adipokines

## Abstract

White adipocytes release adipokines that influence metabolic and vascular health. Hypertrophic obesity is associated with adipose tissue malfunctioning, leading to inflammation and insulin resistance. When pancreatic islet β cells can no longer compensate, the blood glucose concentration rises (hyperglycemia), resulting in type 2 diabetes. Hyperglycaemia may further aggravate adipose cell dysfunction in ~90% of patients with type 2 diabetes who are obese or overweight. This review will focus on the effects of high glucose levels on human adipose cells and the regulation of adipokines.

## 1. Introduction

The mature white adipocyte is distinguished morphologically by the large lipid droplet that occupies most of its interior space. Here, excess energy can be safely stored as an endogenous fuel until metabolic demand requires it to be released. Insulin levels are low in the fasting state, and rise in the post-prandial period. Counter-regulatory hormones, such as epinephrine and glucagon, follow the opposite pattern. Together, they regulate the enzymatic machinery within the adipocyte to achieve the storage of calories as triglyceride following meals, and the release of energy in the form of free fatty acids during fasting or exercise. These well-orchestrated processes maintain metabolic health [1]. The recent recognition of brown/beige adipose tissue in humans has stimulated new avenues of investigation to determine its role in human physiology and pathophysiology, but this topic is beyond the scope of this review.

Adipocytes are also recognized for the myriad of adipokines (cytokines) they produce and release. These bioactive factors have far-ranging effects on many physiological systems including appetite, energy expenditure, insulin sensitivity, inflammation, and coagulation. Immune cells, such as macrophages, take up residence within adipose tissue, and thereby contribute to the overall state of inflammation and functionality within the tissue [2]. Innovative terms to recognize this have entered the lexicon, such as immunometabolism and metabolic inflammation.

The adipocyte population is dynamic, undergoing a cellular turnover of ~10% of cells/year via the death of old cells and the formation of new adipocytes [3]. Stromal preadipocytes (also called adipose progenitor cells or adipose stem cells), when cued appropriately, are able to differentiate from fibroblast-like cells to increase the number of adipocytes [4]. These cells have also been explored as possible regenerative cell therapeutics, as they have the capacity to differentiate not only into adipocytes, but into other mesynchymal lineages, such as bone and cartilage [5].

Healthy remodeling ensures the normal function of the adipose cells [6]. When caloric intake chronically exceeds energy expenditure, adipose tissue expands to accommodate the excess energy. If adipocyte differentiation is not sufficient during this time to produce enough new adipocytes, existing adipocytes will undergo hypertrophy and become dysfunctional, exhibiting inflammation and insulin resistance [7,8].

Overweight/obesity with adipose hypertrophy increases the risk of developing type 2 diabetes. Indeed, a large majority of patients with type 2 diabetes are overweight/obese [9]. The defining metabolic disruption of hyperglycaemia can be considered as a nutrient stress condition.

When human adipocytes undergo nutrient stress due to high glucose levels [10,11], endoplasmic reticulum (ER) function is impaired, and the unfolded protein response (UPR) is activated [12]. In short, the UPR consists of activation of three transmembrane proteins: PKR-like eukaryotic initiation factor 2α kinase (PERK), inositol-requiring enzyme-(IRE) 1α, and activating transcription factor (ATF)-6. PERK and IRE-1α complex with each other, and ATF-6 is processed to an active transcription factor. The widespread effects include increases in BiP/GRP78, calnexin, protein disulfide isomerase, X-BP1, as well as an increase in the phosphorylation of eIF2α, that increases ATF-4 protein and its action on target genes CCAAT/enhancer-binding protein homologous protein (CHOP) and ER oxidoreductin. Chronically, this induces inflammation and insulin resistance e.g., via JNK activation by IRE-1α, and via activation of the inhibitor of IκB kinase (IKK)β-nuclear factor (NF) κB pathway to up-regulate adipokine expression and release e.g., tumour necrosis factor (TNF)α, interleukin (IL)-6, and C-C motif chemokine ligand 2 (CCL2; also known as monocyte chemotactic protein 1).

JNK activation induces insulin resistance within the adipocyte primarily through inhibitory serine/threonine phosphorylation of insulin receptor substrate (IRS). This impairs their ability to be tyrosine phosphorylated by insulin receptors and thus they are less efficient in transmitting the insulin signal further downstream [2]. The pro-inflammatory cytokines induced by NF-kB act though a variety of molecular mechanisms, including the activation of other cellular serine/threonine kinases that interfere with the propagation of the insulin signal [13].

Several other cellular processes, in addition to ER stress, may be perturbed by high glucose conditions to contribute to adipocyte dysfunction. The formation of advanced glycation end-products is increased, and these molecules may limit insulin signaling, e.g., via methylglyoxal binding to IRS that limits IRS tyrosine phosphorylation [14]. High glucose conditions will increase the flux into the hexosamine-synthesis pathway, leading to inhibitory modifications of IRS and insulin receptor by *O*-GlcNAcylation [15]. High glucose conditions increase generation of reactive oxygen species ROS in adipocytes via the mitochondrial electron transport chain [16,17]. The high flux of electrons through mitochondria directs them to coenzyme Q which transfers them to oxygen, forming superoxide and then peroxide by the action of superoxide dismutase. High levels of ROS can activate protein kinase C, a serine/threonine kinase that inhibits insulin signaling. Finally, there is evidence that autophagy, normally protective for cell survival, is excessively up-regulated in adipose tissue in the setting of obesity and type 2 diabetes [18]. This maladaptive response is associated with increased apoptotic markers.

Therefore, high glucose levels may act through multiple mechanisms to alter adipose cell functions. This review will focus on recent studies examining preadipocyte responses, adipocyte differentiation, and adipokine regulation, using human adipose cells. Most of these studies used an elevated value of 15–25 mM glucose (some with dose-response studies) in comparison to a normal glucose concentration of 5 mM.

## 2. High Glucose Levels and Preadipocyte Responses

Dentelli et al. reported that visceral (intra-abdominal) preadipocytes isolated from patients with type 2 diabetes were more numerous than those from control subjects, and that they exist in a more de-differentiated versus a committed state, based on a higher expression of Oct4 and Nanog (indicators of stemness) [19]. When preadipocytes from non-diabetic controls were placed in high glucose levels, Oct4 and Nanog expression increased. High glucose levels also stimulated the rate of preadipocyte proliferation. The generation of ROS by NADPH oxidase was required for these effects. These authors suggested that high glucose levels might favour an abundance of preadipocytes in adipose tissue, which could be accessed relatively easily and has the potential to be used for regenerative cell therapeutic applications. However, as recently noted, the promise of stem cells as novel treatments must be tempered by the need to more fully understand their behaviour and to assess safety risks [20].

Cheng et al. studied subcutaneous preadipocytes from patients with type 2 diabetes, and also found evidence of enhanced stemness, as indicated by a similar upregulation of Oct4 and Nanog, as well as an elevated rate of ROS production [21]. However, in this case, the proliferation phenotype was different than observed by Dentilli et al. [19]. When subcutaneous preadipocytes from control subjects were placed in high glucose levels, the preadipocytes exhibited reduced proliferation and migration. They also displayed increased senescence. One possibility for the difference might be features specific for visceral versus subcutaneous preadipocytes [1].

Gu et al. examined abdominal subcutaneous preadipocytes from patients with type 2 diabetes, and detected lower expression of vascular endothelial growth factor and higher hepatocyte growth factor expression versus control subjects [22]. Proliferation rates of preadipocytes from diabetic versus control subjects were not different under normoxic conditions. However, when placed under hypoxic conditions, a reduction in the proliferation response of preadipocytes from the diabetic patients was observed.

Cramer et al. studied subcutaneous preadipocytes from patients with type 2 diabetes [23]. The proliferation rate was the same for both types of preadipocytes, and was reduced equally by high glucose conditions. Rates of senescence and apoptosis were higher for preadipocytes from the diabetic patients.

Verrijn Stuart et al. found that hyperglycaemic plasma from type 1 diabetes patients accelerated the rate of proliferation of normal preadipocytes [24]. However, this effect was not seen with high glucose concentrations alone.

## 3. High Glucose Levels and Adipocyte Differentiation

Aguiari et al. examined the effect of high glucose levels and observed a positive effect on adipocyte differentiation. However, the experiments were performed in the absence of the usual adipogenic inducers (dexamethasone, isobutylmethylxanthine, and insulin/insulin-like growth factor 1), and as a consequence, the overall extent of differentiation was very low [25].

Cramer et al. studied preadipocytes from patients with type 2 diabetes and noted a small increase in their adipogenic response versus those from control subjects [23]. In contrast, there was a severe reduction in their osteogenic and chondrogenic responses. This variation suggests a selective effect of type 2 diabetes on different lineage pathways, with implications for possible future tissue engineering applications.

Cheng et al. examined preadipocytes placed in high glucose versus normal glucose levels with an adipogenic induction medium [21]. They did not observe any difference in the response, based on lipid accumulation as assessed by the neutral lipid dye Oil Red O.

When human preadipocytes were differentiated in high glucose (17.5 mM in this case) versus normal glucose levels in the presence of adipogenic inducers, there was no difference in differentiation, as assessed by intracellular triglyceride accumulation [26].

Ronningen et al. found that preadipocytes differentiated to the same extent, whether in normal or high glucose levels [27]. They assessed adipogenesis by lipid accumulation with Oil Red O and by induction of mRNA adipogenic genes.

Peshdary et al. performed a detailed time-course analysis of adipogenesis in high glucose versus normal glucose levels, measuring adipogenic protein expression as well as intracellular triglyceride accumulation [28]. There was an equal differentiation response independent of the glucose level at all time points.

Verrijn Stuart et al. found that hyperglycaemic plasma from type 1 diabetes patients enhanced the extent of adipocyte differentiation of normal preadipocytes [24]. However, high glucose concentrations alone had no effect of adipogenesis.

## 4. High Glucose Levels, Inflammation, and Adipokines

Verrijn Stuart et al. found that in type 1 diabetes patients, plasma levels of adiponectin, leptin, retinal binding protein (RBP)-4, cathepsin S, tissue inhibitor of metalloproteinase-1, chemerin, serum amyloid A-1, plasminogen activator inhibitor-1 (PAI)-1, and CCL2 levels were increased compared to control subjects [24]. No significant differences were found in the other plasma adipokines studied, including IL-1RA, IL-1β, IL-6, IL-10, TNF-α, interferon-γ, macrophage inhibitory factor, IL-8, C-X-C motif ligand (CXCL) 10, γ interferon inducible protein 10, granulocyte-macrophage colony stimulating factor, resistin, thrombopoietin, and adipsin.

Elevations in circulating adipokines were also measured in the experimental context of acutely raising plasma glucose levels with a hyperglycaemic clamp and octreotide to block insulin release [29]. Elevations in IL-6, TNF-α, and IL-18 levels occurred, and these were suppressed in the presence of the antioxidant glutathione.

Plasma adipokines in diabetes arise from non-circulating cells, such as adipocytes and endothelial cells [30]. When abdominal subcutaneous adipose tissue explants were placed in high glucose levels, there was an increase in Toll-like receptor 4 protein levels, a pattern recognition receptor that regulates immunity and inflammation, in the explants and in the isolated adipocytes [31]. The protein levels of the related TNF receptor-associated factor 6 also increased in adipocytes, but not the explants. High glucose levels increased IKKβ protein levels in the explants and adipocytes, with a more minor increase in NF-κB levels only in adipocytes. These data suggest that adipocytes are primed for inflammatory responses under the influence of high glucose levels. However, the authors did not observe any concomitant effect of high glucose levels on IL-6 or TNFα release in this study.

In the context of hyperglycaemia arising from type 1 diabetes, Verrijn Stuart et al. investigated whether elevations in plasma adipokine levels correlated with rates of adipokine release from adipocytes [24]. They first tested the effect on normal adipocytes incubated with plasma from patients with type 1 diabetes versus controls, and noted higher rates of release of CCL2, TNF-α, adiponectin, as well as upward trends for RBP-4 and cathepsin S. In contrast, high glucose levels alone increased CCL2 secretion and lowered adiponectin and RBP4 by adipocytes, without any changes in cathepsin S, chemerin, leptin, or PAI-1. This difference in responses suggests other factors, as yet unidentified, in plasma from diabetes patients are bioactive, in addition to high glucose levels.

Ronningen et al. demonstrated that high glucose levels could up-regulate inflammation response genes in preadipocytes [27]. This pro-inflammatory pattern was accentuated during the adipogenic process. They examined the expression of chemerin, CCL2, IL-1β, CXCL1, CXCL5, and IL-8, and there was a moderate enhancement in the expression of all genes except CXCL5 in the presence of high glucose levels. With high glucose levels, there was a reduction in the trimethylation of histones H3K9 and H3K27, and such decreases in histone post-translational modification generally correlate with an increase in transcript levels. These authors proposed that high glucose levels, through an unknown mechanism, attenuate repressive histone H3 modifications on inflammation response gene regulatory elements. Peshdary et al. did not detect any effect of high glucose levels on adipose cell inflammation during the differentiation process [28]. They assessed mRNA expression of IL-6, CCL2, and IL-1β. In the study by Ronningen et al., preadipocytes from non-obese donors were exposed to HG levels for 21 days versus the 14-day duration in the study of Peshdary et al.

## 5. Concluding Remarks

Hyperglycaemia is a common nutrient stress condition that may alter adipocyte inflammation and adipokine release. This can occur in patients with type 2 diabetes who are typically obese with excess adipose tissue, or in patients with type 1 diabetes, who are usually lean. Research examining the effect of high glucose levels on human adipose cell inflammation has provided some limited insights, but is an under-developed area of investigation. In the studies that have been published, there are inconsistencies with respect to the effects on preadipocytes as well as on adipocytes that have yet to be resolved. Further investigation in this area will increase our understanding of the pathophysiology of adipokine dysregulation induced by hyperglycaemic states. In addition, future studies may elucidate the impact of high glucose levels on preadipocytes as potential cellular therapeutics in the field of tissue engineering, although there remains much to learn about the properties of these cells and the safety of such an application.

## Abbreviations

ATF: activating transcription factor; CCL2: C-C motif chemokine ligand 2; CHOP: CCAAT/enhancer-binding protein homologous protein; ER: endoplasmic reticulum; IKK: IκB kinase; IL: interleukin; IRE: inositol-requiring enzyme; IRS: insulin receptor substrate; NF: nuclear factor; PAI: plasminogen activator inhibitor-1; PERK: PKR-like eukaryotic initiation factor eIF 2α kinase; RBP: retinal binding protein; ROS: reactive oxygen species; TNF: tumor necrosis factor; UPR: unfolded protein response.

## References

[1] Tchernof A., Despres J.P. (2013). Pathophysiology of human visceral obesity: An update. Physiol. Rev..

[2] Hotamisligil G.S. (2017). Inflammation, metaflammation and immunometabolic disorders. Nature.

[3] Spalding K.L., Arner E., Westermark P.O., Bernard S., Buchholz B.A., Bergmann O., Blomqvist L., Hoffstedt J., Näslund E., Britton T. (2008). Dynamics of fat cell turnover in humans. Nature.

[4] Cawthorn W.P., Scheller E.L., MacDougald O.A. (2012). Adipose tissue stem cells meet preadipocyte commitment: Going back to the future. J. Lipid Res..

[5] Locke M., Feisst V., Dunbar P.R. (2011). Human adipose-derived stem cells: Separating promise from clinical need. Stem Cells.

[6] Berry D.C., Jiang Y., Graff J.M. (2016). Emerging roles of adipose progenitor cells in tissue development, homeostasis, expansion and thermogenesis. Trends Endocrinol. Metab..

[7] Heilbronn L., Smith S.R., Ravussin E. (2004). Failure of fat cell proliferation, mitochondrial function and fat oxidation results in ectopic fat storage, insulin resistance and type 2 diabetes mellitus. Int. J. Obes..

[8] Sun K., Kusminski C.M., Scherer P.E. (2011). Adipose tissue remodeling and obesity. J. Clin. Investig..

[9] Bain S.C., Feher M., Russell-Jones D., Khunti K. (2016). Management of type 2 diabetes: The current situation and key opportunities to improve care in the UK. Diabetes Obes. Metab..

[10] Alhusaini S., McGee K., Schisano B., Harte A., McTernan P., Kumar S., Tripathi G. (2010). Lipopolysaccharide, high glucose and saturated fatty acids induce endoplasmic reticulum stress in cultured primary human adipocytes: Salicylate alleviates this stress. Biochem. Biophys. Res. Commun..

[11] Zha B.S., Zhou H. (2012). ER stress and lipid metabolism in adipocytes. Biochem. Res. Int..

[12] Gregor M.F., Hotamisligil G.S. (2007). Adipocyte stress: The endoplasmic reticulum and metabolic disease. J. Lipid Res..

[13] Oh K.J., Lee D.S., Kim W.K., Han B.S., Lee S.C., Bae K.H. (2016). Metabolic adaptation in obesity and type 2 diabetes: Myokines, adipokines and hepatokines. Int. J. Mol. Sci..

[14] Gaens K.H., Stehouwer C.D., Schalkwijk C.G. (2013). Advanced glycation endproducts and its receptor for advanced glycation endproducts in obesity. Curr. Opin. Lipidol..

[15] Boucher J., Kleinridders A., Kahn C.R. (2014). Insulin receptor signaling in normal and insulin-resistant states. Cold Spring Harb. Perspect. Biol..

[16] Brownlee M. (2001). Biochemistry and molecular cell biology of diabetic complications. Nature.

[17] Giacco F., Brownlee M. (2010). Oxidative stress and diabetic complications. Circ. Res..

[18] Kosacka J., Kern M., Kloting N., Paeschke S., Rudich A., Haim Y., Gericke M., Serke H., Stumvoll M., Bechmann I. (2015). Autophagy in adipose tissue of patients with obesity and type 2 diabetes. Mol. Cell. Endocrinol..

[19] Dentelli P., Barale C., Togliatto G., Trombetta A., Olgasi C., Gili M., Riganti C., Toppino M., Brizzi M.F. (2013). A diabetic milieu promotes OCT4 and NANOG production in human visceral-derived adipose stem cells. Diabetologia.

[20] Marks P.W., Witten C.M., Califf R.M. (2017). Clarifying stem-cell therapy’s benefits and risks. N. Engl. J. Med..

[21] Cheng N.C., Hsieh T.Y., Lai H.S., Young T.H. (2016). High glucose-induced reactive oxygen species generation promotes stemness in human adipose-derived stem cells. Cytotherapy.

[22] Gu J.H., Lee J.S., Kim D.W., Yoon E.S., Dhong E.S. (2012). Neovascular potential of adipose-derived stromal cells (ASCs) from diabetic patients. Wound Repair Regen..

[23] Cramer C., Freisinger E., Jones R.K., Slakey D.P., Dupin C.L., Newsome E.R., Alt E.U., Izadpanah R. (2010). Persistent high glucose concentrations alter the regenerative potential of mesenchymal stem cells. Stem Cells Dev..

[24] Verrijn Stuart A.A., Schipper H.S., Tasdelen I., Egan D.A., Prakken B.J., Kalkhoven E., de Jager W. (2012). Altered plasma adipokine levels and in vitro adipocyte differentiation in pediatric type 1 diabetes. J. Clin. Endocrinol. Metab..

[25] Aguiari P., Leo S., Zavan B., Vindigni V., Rimessi A., Bianchi K., Franzin C., Cortivo R., Rossato M., Vettor R. (2008). High glucose induces adipogenic differentiation of muscle-derived stem cells. Proc. Natl. Acad. Sci. USA.

[26] Collins J.M., Neville M.J., Pinnick K.E., Hodson L., Ruyter B., van Dijk T.H., Reijngoud D.J., Fielding M.D., Frayn K.N. (2011). De novo lipogenesis in the differentiating human adipocyte can provide all fatty acids necessary for maturation. J. Lipid Res..

[27] Ronningen T., Shah A., Reiner A.H., Collas P., Moskaug J.O. (2015). Epigenetic priming of inflammatory response genes by high glucose in adipose progenitor cells. Biochem. Biophys. Res. Commun..

[28] Peshdary V., Gagnon A., Sorisky A. (2016). Effect of high glucose concentration on human preadipocytes and their response to macrophage-conditioned medium. Can. J. Diabetes.

[29] Esposito K., Nappo F., Marfella R., Giugliano G., Giugliano F., Ciotola M., Quagliaro L., Ceriello A., Giugliano D. (2002). Inflammatory cytokine concentrations are acutely increased by hyperglycemia in humans: Role of oxidative stress. Circulation.

[30] Pickup J.C., Chusney G.D., Thomas S.M., Burt D. (2000). Plasma interleukin-6, tumour necrosis factor alpha and blood cytokine production in type 2 diabetes. Life Sci..

[31] Youssef-Elabd E.M., McGee K.C., Tripathi G., Aldaghri N., Abdalla M.S., Sharada H.M., Ashour E., Amin A.I., Ceriello A., O’Hare J.P. (2012). Acute and chronic saturated fatty acid treatment as a key instigator of the TLR-mediated inflammatory response in human adipose tissue, in vitro. J. Nutr. Biochem..

